# Analysis of a Biathlon Sprint Competition and Associated Laboratory Determinants of Performance

**DOI:** 10.3389/fspor.2019.00060

**Published:** 2019-11-12

**Authors:** Harri Luchsinger, Rune Kjøsen Talsnes, Jan Kocbach, Øyvind Sandbakk

**Affiliations:** Department of Neuromedicine and Movement Science, Faculty of Medicine and Health Science, Centre for Elite Sports Research, Norwegian University of Science and Technology, Trondheim, Norway

**Keywords:** biathletes, cross-country skiing, gross efficiency, maximal oxygen uptake, rifle shooting

## Abstract

Biathlon is an Olympic winter-sport where cross-country (XC) skiing in the skating technique is combined with rifle shooting. In the biathlon sprint competition for men, three laps of 3.3-km are interspersed with a 5-shot shooting sequence in the prone and standing position. Our purpose was to investigate the contribution from overall XC skiing performance, the performance in different terrain sections and shooting performance to the overall biathlon sprint race performance, as well as the relationship to laboratory-measured capacities obtained during treadmill roller ski skating. Eleven elite male biathletes were tracked by a Global Positioning System (GPS) device and a heart rate (HR) monitor during an international 10-km biathlon sprint competition. Within a period of 6 weeks prior to the competition, physiological responses, and performance during submaximal and maximal treadmill roller skiing were measured. Stepwise multiple regression analysis revealed that XC skiing time, shooting performance, shooting time and range time explained 84, 14, 1.8, and 0.2% of the overall sprint race performance (all *p* < 0.01). Time in uphill, varied, and downhill terrains were all significantly correlated to the total XC skiing time (*r* = 0.95, 0.82, 0.72, respectively, all *p* < 0.05). Percent of maximal HR (HRmax) and rating of perceived exertion (RPE) during submaximal roller skiing, and time-to-exhaustion during incremental roller skiing correlated significantly with overall biathlon sprint race performance and overall XC skiing time (*r* = 0.64–0.95, all *p* < 0.05). In conclusion, XC skiing performance provided greatest impact on biathlon sprint performance, with most of the variance determined by XC skiing performance in the uphill terrain sections. Furthermore, the ability to roller ski with a low RPE and %HRmax during submaximal speeds, as well as time-to-exhaustion during incremental roller skiing significantly predicted biathlon performance. Such laboratory-derived measures may therefore be validly used to distinguish biathletes of different performance levels and to track progress of their XC skiing capacity.

## Introduction

Biathlon is an Olympic winter-sport with two main components; cross-country (XC) skiing in the skating technique combined with 5-shot rifle shooting sequences. For men, the biathlon sprint competition consists of three laps of 3.3 km interspersed with shooting in the prone and standing position, in which each missed shot is penalized by adding a 150-m XC skiing loop (IBU, 2017). Shooting is performed on a 50 m shooting range using 0.22 caliber long rifles weighing >3.5 kg that the athletes carry on their back while skiing, and the circular hit areas are 45 mm in diameter in prone and 115 mm in standing shooting. Thus, success in biathlon demands high aerobic endurance capacity, an efficient skiing technique, as well as rapid and accurate shooting performed directly after high-intensity exercise.

A recent investigation of World Cup performance in biathlon sprint events shows that XC skiing time is the most distinguishing factor for the overall performance (Luchsinger et al., 2018), explaining ~59–65% of the overall performance difference between top-10 results and those finishing among 21–30 in both sexes. Furthermore, ~31–35% of the group-difference was explained by shooting performance (i.e., time spent in the penalty loop due to missed targets), whereas shooting time and range time (i.e., time at the shooting range minus shooting time) together explained only 4–6% of the group-difference in overall biathlon sprint performance. This is supported by the work of Skattebo and Losnegard (2017) where the largest between-athlete variability was found for XC skiing time followed by shooting performance during biathlon sprint races.

Although the scientific understanding of XC skiing demands is relatively well-defined (Sandbakk and Holmberg, 2014, 2017; Losnegard, 2019), a more comprehensive understanding of the demands of XC skiing performance in biathlon is needed. In their review of the scientific literature in biathlon, Laaksonen et al. (2018a) wrote that the forced breaks (when shooting) between bouts of close to maximal intensity skiing is unique in endurance sports. More accurate analyses of the skiing component of biathlon races can be gained by combining wearable Global Positioning Systems (GPS) with heart rate (HR) monitoring during competitions. However, while this methodology has not yet been employed in biathlon events for scientific purposes, the many GPS-based studies performed in XC skiing have revealed that more than 50% of the total race time is spent uphill and that these terrain sections are most performance-differentiating (Andersson et al., 2010; Bolger et al., 2015; Sandbakk et al., 2016b; Solli et al., 2018). Although the physiological demands of the XC skiing component of biathlon competitions are comparable to those seen in XC skiing, biathletes compete only in the skating technique and with a rifle carried on the back that alters the energy cost and kinematical aspects of skiing (Stöggl et al., 2015). In addition, biathletes' pacing strategies need to take into account the important 25–30 s shooting sequences during the competition (Laaksonen et al., 2018b). Therefore, biathletes may use less effort on uphill terrain sections to avoid accumulation of fatigue when approaching the shooting range.

In addition, knowledge about the underlying laboratory-measured performance-determinants for XC skiing performance in modern biathlon sprint races are lacking. XC skiing performance has previously been linked to peak oxygen uptake (VO_2peak_) and the ability to effectively convert metabolic energy into external work rate and speed [i.e., gross efficiency (GE)] in XC skiers (Sandbakk et al., 2010, 2011b, 2013, 2016a,b) and Nordic combined athletes (Sandbakk et al., 2016c; Rasdal et al., 2017). This has provided coaches and athletes with valuable insight into the relationships between competition performance and different performance-indices obtained in the laboratory. However, the current knowledge on the importance of these factors in biathlon is scarce, and their association to performance has not been studied since the mid-1990s (Rundell, 1995; Rundell and Bacharach, 1995).

On this basis, the present study aims to investigate the contribution from overall XC skiing performance, performance in different terrain sections, and shooting performance to the overall biathlon sprint race performance. In addition, we aim to examine the relationships between overall biathlon and XC skiing performance to laboratory-measured capacities obtained during treadmill roller skiing. We hypothesize that XC skiing performance on uphill terrain provides the strongest relationships with overall biathlon sprint performance, and that uphill performance would correlate strongly with VO_2peak_ and gross efficiency while treadmill roller skiing.

## Methods

### Participants

Eleven elite male biathletes, members of the junior and recruitment team of the Norwegian Biathlon Association, competing in the IBU-cup, Jr. World championships and at the highest level in the Norwegian cup, volunteered to participate in the study. The participant's age, anthropometrics and training characteristics are presented in Table 1.

**Table 1:** Characteristics (mean ± SD) of the eleven elite male biathletes participating in the study.

Age (yrs)	21.4 ± 2.1
Body height (cm)	181.1 ± 4.7
Body mass (kg)	76.5 ± 4.8
Body mass index (kg·m^−2^)	23.5 ± 1.3
Rifle weight (kg)	4.0 ± 0.3
Annual training^a^ (hrs)	685 ± 115
Physical training^a^ (hrs)	585 ± 87
Shooting training^a^ (hrs)	100 ± 34
Maximum HR^b^ (beats·min^−1^)	198 ± 8

a *Training volume categorized into hours of total training, physical training and shooting training during the last 12 months prior to the competition*.

b *Self-reported maximum heart rate (HR_max_) based on outdoor tests from the year prior to this study*.

### Ethics Statement

The Regional Committee for Medical and Health Research Ethics waives the requirement for ethical approval for this study. Therefore, the ethics of the study is done according to the institutional requirements and approval for data security and handling was obtained from the Norwegian Centre for Research Data. Prior to the data collection, all participants provided written informed consent to voluntarily take part in the study. The participants were informed that they could withdraw from the study at any point in time without providing a reason for doing so.

### Overall Design

During an international 10-km biathlon sprint competition in mid-November 2016, regulated by the International Biathlon Association (IBU), all study participants were tracked by a GPS device and HR monitor. The racecourse was mapped with a coupled GPS and barometer to provide a valid course and elevation profile. The XC skiing course was further divided into uphill, varied, and downhill terrain sections, and the overall shooting component was separated into range time (time spent at the shooting range excluding shooting time), shooting time and penalty time (time spent in the penalty loop as a consequence of misses at the shooting range). Within a period of 6 weeks prior to the competition, all participants completed submaximal and maximal laboratory testing while roller skiing on a treadmill using different speed and incline combinations.

### Laboratory Testing

Initially, the participants performed 15 min of low-intensity warm-up while roller skiing on the treadmill. The first 10 min of the warm-up were conducted without the rifle and the last 5 min while carrying the rifle on their back. Thereafter, the submaximal tests were performed consisting of two 5-min stages (one with and one without carrying the rifle) with 2-min recovery in-between using each of the three most important sub-techniques (G2–G4) in the skating technique (for a more detailed description of sub-techniques, see Andersson et al., 2010). The first two stages were conducted utilizing the G4 sub-technique at 3% inclination and 20 km·h^−1^, followed by two stages using the G3 sub-technique at 5% inclination and 15 km·h^−1^. The two last stages were performed with the G2 sub-technique at 12% inclination and a speed of 8 km·h^−1^, respectively. The inclines were based on previous research indicating which inclines the different sub-techniques are naturally employed. The speeds were chosen to match all inclines for metabolic cost, based on pilot tests of biathletes and XC skiers in our laboratory. Respiratory variables and HR were measured continuously and the average of the last 2 min of each stage was used for steady-state analyses. Blood lactate concentrations and RPE were determined directly after completing each submaximal stage. In the final analyses, only the measurements using the rifle were used. After a 5-min recovery period, all participants completed maximal roller skiing using an incremental test to exhaustion to determine VO_2peak_ and time to exhaustion (TTE; as a measure of performance). The starting incline and speed was 10% and 11 km·h^−1^. The initial speed was kept constant, while the incline was increased by 2%-points every minute up to 14%. Thereafter, the speed was increased by 1 km·h^−1^ every minute until exhaustion. Respiratory variables and HR were measured continuously and VO_2peak_ was defined as the average of the three highest and consecutive 10 s measurements. Peak HR (HR_peak_) was defined as the highest 5 s HR measurement during the test. Blood lactate concentrations and RPE were measured directly after the maximal-test. Treadmill roller skiing was performed on a 5 × 3 m motor-driven treadmill (Forcelink B.V., Culemborg, The Netherlands) with non-slip rubber surface on the treadmill belt, allowing the participants to use their own poles with special carbide tips. To minimize variations in roller resistance, the participants used the same pair of skating roller skis with standard category 2 wheels (IDT Sports, Lena, Norway). Before the tests, rolling friction force (F_f_) was tested with a towing test as previously described (Sandbakk et al., 2010). The rolling friction coefficient (μ) was determined by dividing F_f_ by the normal force (F_n_ = F_f_ /F_n_), and provided an average μ value of 0.0195, which was included in the calculation of work rate. The biathletes used their own rifle with an average weight of 4.0 ± 0.3 kg during laboratory testing.

Respiratory variables were measured using open-circuit indirect calorimetry with mixing chamber and 30 s averages of the respiratory variables were used (Oxycon Pro, Jaeger GmbH, Hoechberg, Germany). The instruments were calibrated against ambient air conditions and certified gases of known concentrations of O_2_ (15.0%) and CO_2_ (5.0%) before each test session. The flow transducer (Triple V, Erick Jaeger GmbH, Hoechberg, Germany) was calibrated using a 3-L high-precision calibration syringe (Calibration syringe D, SensorMedics, Yorba Linda, CA, USA). HR was continuously measured with a Polar V800 monitor and synchronized with the Oxycon Pro system. Blood lactate in 20 μL of blood was taken from the fingertip and measured using the stationary Biosen C-Line lactate analyzer (Biosen, EKF Industrial Electronics, Magdeburg, Germany). The device was calibrated every 60 min with a 12 mmol·L^−1^ standard concentration. Rating of perceived exertion (RPE) was determined using the 6–20 Borg Scale (Borg, 1982). The participants' body-mass and mass of the rifle were measured using a precise weight (Seca, model 708, GmbH, Hamburg, Germany), and body-height using a calibrated stadiometer (Holtain Ltd, Crosswell, UK), prior to the test.

Work rate was calculated as the sum of power against gravity and friction: P_g_+P_f_ = mgv [sin(α)+cos(α)μ], with P_g_ being power against gravity, P_f_ power against friction, *m* the biathletes body-mass including skiing shoes, roller skis (and the rifle when roller skiing with the rifle), *g* the gravitational constant, α the treadmill incline, μ the frictional coefficient and *v* the treadmill speed. The aerobic metabolic rate was calculated as the product of VO_2_ and the oxygen energetic equivalent using the associated respiratory exchange ratio and standard conversion tables (Peronnet and Massicotte, 1991). GE was defined as the ratio of work rate and aerobic metabolic rate and calculated from the submaximal tests (Sandbakk et al., 2010).

### Competition Analysis

**Figure 1:**
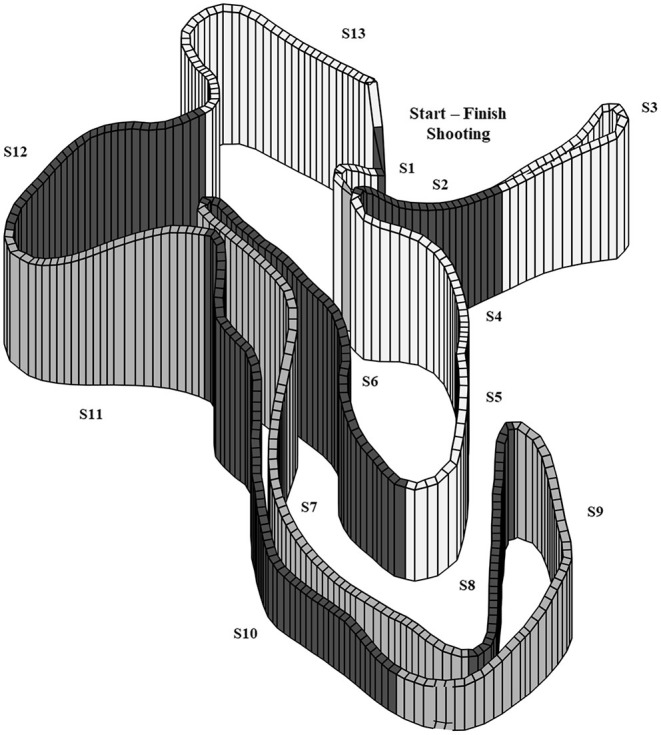
3D illustration of the 3015 m XC skiing racecourse divided into 13 different terrain sections. Detailed information about the terrain sections is described in Table 3.

Prior to the competition, all the participants completed low-intensity warm-up procedures according to their own optimized protocols used in both training and competition. All participants used their own equipment during the competition, including rifle (4.0 ± 0.3 kg), poles (91 ± 1% of body height), skating XC skiing shoes, and skating XC skis. The skis were accustomed to individual preferences and prepared for the current conditions with appropriate ski base material and chamber stiffness. The weather conditions were stable throughout the entire competition with average ambient air and snow temperatures of −5.5 and −7.5°C, respectively. The average relative humidity was 85% during the competition and the wind was low and stable at the shooting range, varying between 0.3 and 1.0 m/s. Weather conditions were continuously registered during the competition using a weather station developed by the Norwegian Top Sport Centre (delivered by Airtight Ltd., Oslo, Norway), measuring both air and snow temperatures and humidity. Wind speeds during the competition were collected from the official shooting results (Biathlon, 2017). The racecourse consisted of a combination of artificial and natural snow and was machine-groomed the same morning as the competition day. The course was set in an open area with minimal tree cover and no steep mountains that could interfere with the GPS signals. Course and elevation profiles of the racecourse were measured with an integrated GPS and barometry using a Garmin Forerunner 920 XT (Garmin Ltd., Olathe, Kansas, USA), which collected position and altitude data at a 1 Hz sampling rate to define a reference course with accompanying altitude profile as previously described by Bolger et al. (2015) and Sandbakk et al. (2016b). The participants hit rate (number of targets hit) was provided by the official competition shooting results collected with an electronic target system (Megalink) (Biathlon, 2017). Penalty time was used as the measure of shooting performance in multiple regression and correlation analyses. The XC skiing course was divided into uphill, varied, and downhill terrain that equaled 37, 29, and 34% of the total course distance, respectively. A part of the XC skiing course prior to the shooting range was defined for analysis of pacing toward shooting. This part of the course included terrain section 12, defined as uphill, and section 13, defined as varied terrain (Figure 1, **Table 3**). In addition, each participant's time spent in this part toward shooting was divided by the total XC skiing time in uphill and varied sections on each lap for analyses of their relative time in this “preparation phase prior to shooting” compared to the rest of the course. The classification of different terrain sections was based on the International Ski Federation (FIS) homologation manual for XC skiing racecourses (FIS, 2017). A section boundary was defined where a change between positive and negative gradient in the XC skiing course profile occurred. Terrain sections with climb >10 m and gradient >6% were classified as uphill sections. Sections with descent >10 m and negative gradient >6% were classified as downhill sections. Remaining sections were classified as varied terrain, including short uphill and downhill sections interspersed with flat sections. A part of the racecourse, consisting of flat terrain in the start and finish of the competition, was not included in the final analysis to ensure that the start and final sprint would not affect the analyses of pacing strategies. The exact distance for each lap of the XC skiing racecourse was then 3,015 m.

During the competition, each participant was tracked by a Polar V800 GPS (Polar Electro Oy, Kempele, Finland), which collected position and HR data at a 1 Hz sampling rate. All GPS watches were turned on at least 30 min before the start of the race to ensure that the GPS watches could acquire contact with as many satellites as possible before race start, in order to optimize GPS accuracy for the duration of the race. Furthermore, data for all the participants were adapted to the reference course by fitting each competitors' GPS track to points along the reference course. This method, developed in cooperation between the national biathlon and XC ski federations, Norwegian Olympic Sports Centre and academic institutions provide sufficiently accurate data for the analyses needed here, amounting to a measurement error of up to ±1 s for each 180 m-split, when being compared to more accurate GPS-systems (Gløersen et al., 2018). Virtual split times were defined at every section boundary (uphill, downhill, varied terrain) along the course. Virtual split times in the shooting component were defined using a combination of GPS position and speed data. The time each participant spent in the different components of the race, as well as HR characteristics were calculated based on these virtual split times. Shooting time was defined as the time on the shooting range when speed was below 1.8 m/s (when athletes were at the shooting mat), whereas penalty time was defined as the time spent between a point after the range (i.e., before the penalty loop) and a point after the penalty loop. Thus, athletes with no mistakes also had a short penalty time. Range time was defined as the time spent at the shooting range, without shooting time.

### Statistical Analysis

All data were tested for normality using a Shapiro–Wilk test in combination with visual inspection of data, and all variables are presented as mean ± SD. Correlations between overall biathlon sprint race performance and the different sections of the race, as well as correlations to laboratory capacities, were calculated using the Pearson's product-moment correlation coefficient or with the non-parametric Spearman's rank in cases where data were not normally distributed. Deviation from normally distributed data only occurred in the case of blood lactate concentration during submaximal testing of the three sub-techniques. In these cases, the Spearman's rank test was applied. The coefficient of variation (CV = standard deviation/mean) of time in different terrain sections of the XC skiing racecourse was calculated. Differences between prone and standing position with respect to shooting time and HR, as well as differences in pacing between laps, were tested using the paired sample *t*-test procedure. In addition, we performed two different stepwise multiple regression analyses, with model 1 having overall biathlon performance as dependent variable and XC skiing performance, shooting performance, shooting time, and range time as independent variables. In model 2, XC skiing performance was the dependent variable, and time in different terrains were independent variables. Alpha values of <0.05 determined the level of statistical significance and alpha values between 0.05 and 0.1 were considered trends. All statistical analyses were performed using IBM SPSS Software for Mac, Version 21.0 (SPSS Inc., Chicago, IL).

## Results

### Overall Biathlon Sprint Race Performance

**Figure 2:**
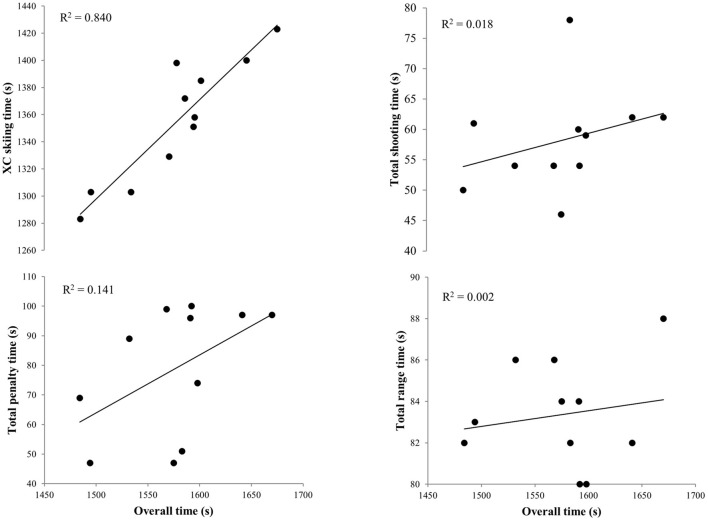
Overall competition time in relationship to XC skiing time, penalty time, shooting time, and range time during a biathlon sprint competition among 11 elite male biathletes. Presented with individual data points and trend lines based on linear regression.

The distribution of XC skiing time, penalty time, shooting time, and range time in the overall biathlon sprint race time was 86.0, 5.0, 4.0, and 5.0%, respectively (Table 2). Stepwise multiple regression analysis demonstrated that XC skiing time explained 84.0% (semi-partial *R*^2^ = 0.603), penalty time 14.2% (semi-partial *R*^2^ = 0.139), shooting time 1.8% (semi-partial *R*^2^ = 0.020), and range time 0.2% (semi-partial *R*^2^ = 0.002) of overall sprint race time variance (all *p* < 0.01). In addition, XC skiing time was significantly correlated to overall biathlon sprint race performance (*r* = 0.92, *p* < 0.01; Figure 2).

**Table 2:** Overall performance, shooting performance, and time spent in the different components of a biathlon sprint competition among eleven elite male biathletes (mean ± SD).

Overall time (s)	*1, 574*±52		
XC skiing time (s)	*1, 355*±43		
Overall shooting component (s)	219 ± 23		
Terrain sections (s)	**Uphill**	**Varied**	**Downhill**
	701 ± 31	339 ± 11	315 ± 6
**Shooting performance**	**Prone**	**Standing**	**Total**
Hit rate (%)	91 ± 7	86 ± 6	89 ± 9
Penalty time (s)	34 ± 15	44 ± 15	78 ± 21
Shooting time (s)	31 ± 5	27 ± 4	58 ± 8
Range time (s)	41 ± 1	42 ± 1	83 ± 2

### XC Skiing Performance

Time, CVs of time and speed in different terrain sections of the XC skiing course are presented in Table 3. The distribution of total XC skiing time spent in uphill, varied, and downhill terrains were 52.0, 25.0, and 23.0%, respectively. Stepwise multiple regression analysis demonstrated that time in uphill explained 90.7% (semi-partial *R*^2^ = 0.315), varied 8.6% (semi-partial *R*^2^ = 0.023) and downhill terrain 0.7% (semi-partial *R*^2^ = 0.007) of the total variation in overall XC skiing time (all *p* < 0.01). Time in uphill, varied, and downhill terrains were all significantly correlated to the overall XC skiing time (*r* = 0.95, 0.82, 0.72, respectively, all *p* < 0.05), and highest CVs of time were found in uphill terrain sections.

**Table 3:** Length (for each 3-km lap), elevation, time and speed, as well as coefficient of variance (CV) of time within different sections of terrain during the three laps of the sprint competition among eleven elite male biathletes.

				**Lap 1**			**Lap 2**			**Lap 3**		
**Section number**	**Terrain type**	**Section length (m)**	**Elevation (m/%)**	**Mean section time (s)**	**Time CV (%)**	**Mean section speed (m/s)**	**Mean section time (s)**	**Time CV (%)**	**Mean section speed (m/s)**	**Mean section time (s)**	**Time CV (%)**	**Mean section speed (m/s)**
S1	Varied^a^	45	–	9 ± 1	5.3	4.8	13 ± 2	13.6	3.4	13 ± 2	12.8	3.5
S2	Downhill	128	14/11	14 ± 1	6.2	9.5	14 ± 1	5.6	9.2	15 ± 1	7.3	8.9
S3	Varied^a^	226	–	27 ± 1	3.3	8.3	29 ± 1	4.4	7.8	30 ± 1	4.4	7.7
S4	Uphill	125	11/9	22 ± 1	5.2	5.6	24 ± 1	4.6	5.2	23 ± 2	6.7	5.4
S5	Varied^a^	304	–	36 ± 2	4.4	8.4	38 ± 2	4.5	8.0	38 ± 2	6.0	8.1
S6	Uphill	279	18/7	55 ± 3	5.3	5.0	58 ± 3	5.6	4.8	57 ± 4	6.9	4.9
S7	Downhill	428	27/6	41 ± 1	3.1	10.4	42 ± 1	2.8	10.2	43 ± 1	3.0	10.0
S8	Uphill	183	14/7	32 ± 2	5.8	5.8	33 ± 3	7.5	5.5	33 ± 2	6.6	5.6
S9	Downhill	288	18/6	30 ± 1	4.2	9.6	30 ± 1	3.4	9.6	31 ± 1	4.0	9.4
S10	Uphill	363	31/9	85 ± 6	6.6	4.2	87 ± 6	6.7	4.2	84 ± 7	8.1	4.3
S11	Downhill	178	15/8	19 ± 1	2.8	9.6	19 ± 1	3.4	9.4	19 ± 1	4.0	9.4
S12	Uphill	179	14/18	36 ± 1	4.0	5.0	37 ± 1	4.2	4.9	34 ± 2	7.1	5.2
S13	Varied^a^	289	–	35 ± 1	1.8	8.3	36 ± 1	2.4	8.1	35 ± 1	3.4	8.3
Sum	Varied	864	–	107	3.7	7.5	116	6.2	6.8	116	6.7	6.9
Sum	Uphill	1,129	88	230	5.4	5.1	239	5.7	4.9	231	7.1	5.1
Sum	Downhill	1,022	74	104	4.1	9.8	105	3.8	9.6	108	4.6	9.4
Total		3,015	118^b^	441 ± 14	3.2	6.8	460 ± 16	3.5	6.5	454 ± 20	4.4	6.6

a *Elevation is not provided for varied terrain sections since these parts of the course consist of small uphills and downhills, as well as flat sections. For detailed specifications of varied terrain, see the Methods section and Figure 1*.

b *Total climb in one lap*.

### Shooting Performance

The average hit rate was 89 ± 9% with 91 ± 7% in the prone position, which was significantly better than the 86 ± 6% in the standing position (*p* < 0.05). On average, %HR_max_ was 87 ± 3% at the start of the shooting, both in the prone and standing position. During shooting, HR_max_ decreased to 69 ± 6% in the prone position, whereas this drop was significantly smaller, decreasing to 79 ± 4%, in the standing position (all *p* < 0.01). On average, the biathletes in this study shot 4 s (13%) faster in the standing than in prone position (*p* < 0.05). There was no significant relationship observed between the biathletes %HR_max_ at the start of shooting and shooting performance, in either prone or standing shooting.

### Pacing Strategies and Skiing Speed Toward Shooting

**Figure 3:**
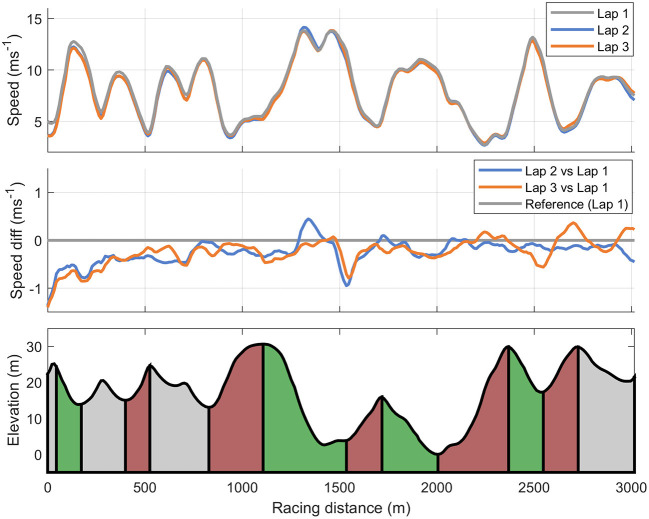
Cross-country skiing speed for each of the three 3-km laps **(upper panel)** and speed differences on lap 2 and 3 compared to the first lap **(mid panel)** during a biathlon sprint competition among 11 elite male biathletes.

**Figure 4:**
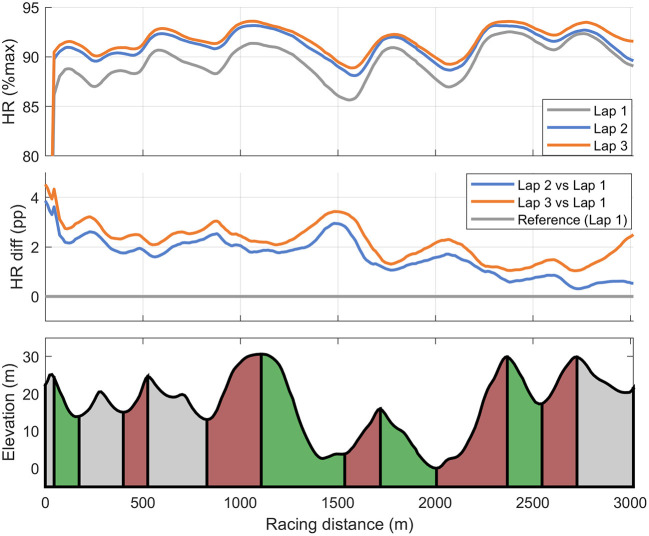
Cross-country skiing heart rate (%HR_max_) for each of the three 3-km laps **(upper panel)** and heart rate differences [in percent points (pp)] on lap 2 and 3 compared to first lap **(mid panel)** during a biathlon sprint competition among 11 elite male biathletes.

Speed and HR profiles for the three laps are shown in Figures 3, 4. Lap times (details provided in Table 3) on all three laps were significantly correlated to the overall XC skiing performance (*r* = 0.84, 0.95, 0.85, *p* < 0.01). The second and third laps were skied with 4.4 and 2.9% slower speeds in comparison to the first lap, respectively, with the last lap being significantly faster than the second lap (all *p* < 0.05). The participant's mean time in the defined section prior to shooting was 71 ± 2 and 73 ± 2 s for the prone and standing position, respectively. However, relative times in this section compared to total time in uphill and varied sections in the rest of the course on each lap were 0.7 ± 0.9%—points faster on the second and 3.1 ± 2.0%—points faster on the third lap compared to the first lap (*p* < 0.05). In addition, relative time in the section prior to shooting was inversely correlated to total time on lap 2 (*r* = −0.64, *p* < 0.05) and lap 3 (*r* = −0.75, *p* < 0.01) but not on lap 1 (*r* = −0.49, *p* = 0.13). There was no significant correlation observed between absolute or relative time or intensity in the last section before shooting and shooting performance (i.e., penalty time) in the prone or the standing position.

### Laboratory Determinants of XC Skiing Performance

**Figure 5:**
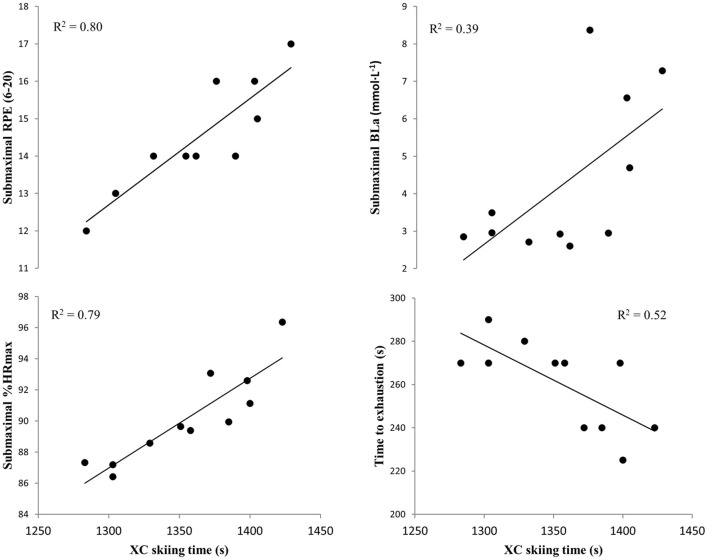
Cross-country (XC) skiing time in relationship to rating of perceived exertion (RPE), relative heart rate (%HR_max_), blood lactate concentrations (BLa) during submaximal roller skiing using the G2 sub-technique, and time to exhaustion during maximal roller skiing among 11 elite male biathletes. Presented with individual data points and trend lines based on linear regression.

Results from laboratory testing is provided in Table 4. During submaximal roller skiing, RPE and %HR_max_ for G2 and G3 sub-techniques were significantly correlated (Figure 5, all *p* < 0.05), and tended to correlate for the G4 technique (both RPE and %HR_max_ 0.06 < *p* < 0.07) with overall biathlon sprint race performance, XC skiing time and time spent in all terrains (Table 5). Furthermore, blood lactate concentrations in the G3 and G2 sub-techniques were significantly correlated to overall XC skiing time and time spent in downhill terrain sections (all *p* < 0.05) and showed a trend to time spent in uphill terrain (*p* = 0.07 and *p* = 0.10 for G2 and G3, respectively).

**Table 4:** Submaximal and maximal physiological responses and treadmill performance (mean ± SD) while roller skiing using different sub-techniques with (R) and without (N) the rifle on the back among eleven elite male biathletes.

**Submaximal tests**	**G4R**	**G4N**	**G3R**	**G3N**	**G2R**	**G2N**
VO_2_ (L·min^−1^)	4.45 ± 0.28	4.24 ± 0.28**	4.44 ± 0.24	4.20 ± 0.23**	4.40 ± 0.26	4.20 ± 0.24**
VO_2_ (mL·min^−1^·kg^−1^)	57.9 ± 2.8	55.3 ± 2.6**	57.7 ± 2.0	54.6 ± 1.8**	57.3 ± 2.4	54.6 ± 2.4**
VO_2_ in % VO_2peak_	79 ± 5	75 ± 4**	78 ± 4	74 ± 4**	78 ± 4	74 ± 4**
RER	0.95 ± 0.02	0.94 ± 0.03	0.94 ± 0.03	0.93 ± 0.03^*^	0.93 ± 0.03	0.91 ± 0.03**
HR (beats·min^−1^)	176 ± 8	171 ± 7**	178 ± 8	174 ± 8**	178 ± 7	174 ± 7**
HR in %HR_max_	89 ± 3	87 ± 2**	90 ± 3	88 ± 3**	90 ± 3	88 ± 3**
RPE (6–20)	14 ± 1	13 ± 1**	14 ± 1	13 ± 1**	15 ± 1	14 ± 1**
BLa (mmol·L^−1^)	3.8 ± 1.3	3.3 ± 1.0**	4.3 ± 1.9	3.7 ± 1.7**	4.3 ± 2.0	4.0 ± 1.8**
GE (%)	14.6 ± 0.7	14.6 ± 0.8	15.4 ± 0.5	15.6 ± 0.6	16.7 ± 0.7	16.7 ± 0.7
**VO**_**2peak**_**-test**
VO_2peak_ (L·min^−1^)				5.63 ± 0.41		
VO_2peak_ (mL·min^−1^·kg^−1^)				73.7 ± 3.9		
Peak RER				1.12 ± 0.30		
Peak HR (beats·min^−1^)				193 ± 8		
Peak BLa (mmol·L^−1^)				13.5 ± 1.3		
RPE (6-20)				19 ± 1		
TTE (s)				260 ± 20		

VO_2_, oxygen uptake; HR, heart rate; HR_max_, maximal heart rate based on outdoor tests from the year prior to this study; RPE, rating of perceived exertion; BLa, blood lactate concentration; GE, gross efficiency; VO_2peak_, peak oxygen uptake from incremental test to exhaustion; TTE, time to exhaustion;

* Significant difference between with and without rifle within sub technique (**p < 0.01,*p < 0.05).

**Table 5:** Correlations between physiological and performance variables obtained during submaximal and maximal roller skiing and the different XC skiing components among 11 elite male biathletes.

	**Total time (s)**	**XC time (s)**	**Uphill terrain (s)**	**Varied terrain (s)**	**Downhill terrain (s)**
**Submaximal G4**
VO_2_ (L·min^−1^)	−0.07	0.07	0.17	−0.27	0.17
VO_2_ (mL·min^−1^·kg^−1^)	0.05	0.16	0.16	−0.02	0.34
VO_2_ in % VO_2peak_	0.26	0.29	0.25	0.13	0.55
HR (beats·min^−1^)	0.28	0.07	−0.05	0.34	0.15
HR in % of HR_max_	0.91^**^	0.89^**^	0.87^**^	0.71^*^	0.58
RPE (6-20)	0.81^**^	0.93^**^	0.95^**^	0.64^*^	0.57
BLa (mmol·L^−1^)	0.45	0.49	0.43	0.35	0.63^*^
GE (%)	−0.05	−0.11	−0.15	0.09	−0.19
**Submaximal G3**
VO_2_ (L·min^−1^)	−0.00	0.17	0.28	−0.21	0.12
VO_2_ (mL·min^−1^·kg^−1^)	0.18	0.30	0.34	0.11	0.33
VO_2_ in % VO_2peak_	0.40	0.43	0.39	0.26	0.58
HR (beats·min^−1^)	0.31	0.14	0.00	0.37	0.27
HR in % of HR_max_	0.85^**^	0.89^**^	0.87^**^	0.67^*^	0.69^*^
RPE (6-20)^b^	0.76^**^	0.84^**^	0.82^**^	0.58^*^	0.71^*^
BLa (mmol·L^−1^)	0.51	0.61^*^	0.53	0.48	0.78^**^
GE (%)	−0.19	−0.31	−0.37	−0.07	−0.21
**Submaximal G2**
VO_2_ (L·min^−1^)	0.02	0.29	0.37	−0.07	0.28
VO_2_ (mL·min^−1^·kg^−1^)	0.19	0.46	0.44	0.26	0.54
VO_2_ in % VO_2peak_	0.39	0.53	0.47	0.36	0.72^*^
HR (beats·min^−1^)	0.27	0.13	−0.01	0.37	0.30
HR in % of HR_max_	0.80^**^	0.89^**^	0.85^**^	0.67^*^	0.73^*^
RPE (6-20)	0.80^**^	0.90^**^	0.86^**^	0.70^*^	0.70^*^
BLa (mmol·L^−1^)	0.46	0.63^*^	0.56	0.46	0.75^**^
GE (%)	−0.21	−0.48	−0.50	−0.23	−0.44
**Maximal roller skiing**
VO_2peak_ (L·min^−1^)	−0.25	−0.11	−0.01	−0.30	−0.16
VO_2peak_ (mL·min^−1^·kg^−1^)	−0.22	−0.16	−0.12	−0.14	−0.30
TTE (s)	−0.67^*^	−0.72^*^	−0.56	−0.75^**^	−0.85^**^

*VO_2_, oxygen uptake; HR, heart rate; HR_max_, maximal heart rate based on outdoor tests from the year prior to this study; RPE, rating of perceived exertion; BLa, blood lactate concentration; GE, gross efficiency; VO_2peak_, peak oxygen uptake from incremental test to exhaustion; TTE, time to exhaustion*.

* *p < 0.05*,

** *p < 0.01*.

The observed correlations between submaximal roller skiing and XC skiing performance did not change when using measurements from roller skiing without the rifle, although carrying a rifle was associated with a 5% higher submaximal oxygen cost, 3% higher HR, ~0.5 mmol/L higher blood lactate concentrations and 7% higher ratings of perceived exertion in comparison to skiing without a rifle (all *p* < 0.01). GE was unchanged, and the observed differences in GE between skiing with and without rifle were independent of sub-technique utilized at the different speeds and inclines (see details in Table 4).

TTE during maximal roller skiing correlated significantly with overall biathlon sprint race time, XC skiing time, as well as time spent in varied and downhill terrain (Table 5; all *p* < 0.05), whereas TTE tended to correlate with time in uphill terrain (*p* = 0.07). In addition, TTE did not correlate with XC skiing performance on the first or second lap but correlated strongly with the 3rd lap (*r* = −0.84, *p* < 0.01).

## Discussion

The primary aim of the present study was to investigate the contribution from XC skiing and shooting performance to the overall biathlon sprint performance, as well as the relationship to laboratory-measured capacities obtained during treadmill roller skiing. The main findings from the stepwise multiple regression analyses showed that XC skiing time explained 84% of the overall biathlon sprint performance, with shooting performance (i.e., penalty time) explaining 14% of the remaining variance and shooting time and range time together explaining the remaining 2%. Time spent in uphill terrain sections had the strongest impact on XC skiing performance (and explained 91% of the variance), although performance in all types of terrain showed significant associations. In addition, RPE and %HR_max_ during submaximal roller skiing as well as TTE during incremental treadmill roller skiing in the laboratory were significantly correlated to overall biathlon performance and isolated XC skiing performance.

### Overall Biathlon Sprint Race Performance

In the current study, XC skiing performance was clearly the most important contributor to the overall biathlon sprint performance, which support previous studies on biathlon sprint races (Skattebo and Losnegard, 2017; Luchsinger et al., 2018). In our approach, the stepwise multiple regression analysis demonstrated that XC skiing performance explained 84% of the variation in overall performance, while only 16% of the remaining variation in performance was explained by the overall shooting component (including shooting performance, shooting time, and range time). Furthermore, the correlation between XC skiing and overall performance was clearly larger than the corresponding correlations for the overall shooting component. These main findings extend upon the recent findings by Luchsinger et al. (2018) who revealed XC skiing time to be the most important contributor to the overall performance difference between top-10 results and those finishing among 21–30 in both sexes in biathlon World Cup sprint races. In the study by Luchsinger et al. (2018), XC time explained ~60% of the variance in overall performance when averaged over 47 World Cup sprint races. The larger influence of XC skiing time found here may be due to different methodologies between the studies, as well as natural variations across competitions (e.g., racecourses with different terrain in the sections prior to shooting and variation in snow and weather conditions). In our case, the shooting conditions were good (i.e., low wind speeds and good visibility) and athletes had relatively high hit rates, similar to the 92–93% hit rates reported among top-10 racers in World Cup sprint races (Luchsinger et al., 2018). Thus, these factors could additionally have contributed to the high impact of XC skiing performance on the overall biathlon sprint performance in the current study. However, the previous studies also highlight the larger importance of XC skiing performance than shooting performance to the overall biathlon sprint performance. Extending upon these findings, the current study provides detailed insight into the different components of the biathlon sprint competition, such as the importance of XC skiing in different terrains and the effects of pacing strategy.

### XC Skiing Performance

The relative distribution of time spent in the uphill terrain sections accounted for 52% of the total XC skiing time and, additionally, time spent uphill revealed a near perfect correlation with XC skiing time. These findings are supported by higher CVs of time within the uphill compared to varied and downhill terrain sections, indicating greatest variation in time spent uphill, followed by time spent in the varied and downhill terrain sections, respectively. Altogether, the stepwise multiple regression analysis demonstrated that time in uphill explained ~91% of the variation in XC skiing performance. Indeed, uphill terrain as the most performance-differentiating part of the XC skiing performance is supported by previous research in XC skiing (Andersson et al., 2010; Bolger et al., 2015; Sandbakk et al., 2016b; Solli et al., 2018). However, in line with findings from XC skiing (Sandbakk et al., 2016b), performance in all types of terrain are important for achieving an excellent XC skiing performance in biathlon as shown by the significant correlations between all types of terrain and isolated skiing performance in this biathlon sprint race.

In line with findings from Biathlon World Cup sprint races (Luchsinger et al., 2018), the biathletes reduced their speed during the second and third lap compared to the first lap of the race, but skied the third lap faster compared to the second lap. This indicates that athletes maintain physical reserves to increase speed during the latter part of the race. Specifically, the slightly reduced speed during the second lap prior to shooting in the standing position may be a strategy to minimize a possible negative effect of exercise intensity on shooting performance. This assumption is strengthened by findings in XC skiing where the athletes (who do not stop to shoot during the race) employ a more clear positive pacing strategy (although with a short end spurt) (Bolger et al., 2015; Losnegard et al., 2016; Sandbakk et al., 2016b). Extending upon the findings from World Cup sprint races, the detailed race analysis in this study shows that the time in the last section prior to shooting relative to the overall lap time was inversely correlated to total XC skiing time on lap 2 and 3. This means that faster skiers generally employed higher speed through the entire laps 2 and 3, but that they paced slower toward standing shooting compared to slower skiers. This pacing strategy fits well to the fact that biathletes miss more in the standing position than in prone. It also means that slower biathletes should carefully evaluate race tactics concerning the approach to the shooting range. A possible limitation of our approach is the use of a GPS watch with 1-Hz sampling frequency that has some accuracy limitations in comparison to high-end GNSS-systems (e.g., a 10 Hz standalone GNSS receiver or a differential GNSS; Gløersen et al., 2018). However, attaching standalone high-accuracy GNSS-units onto the biathletes was not an option in this case, as we have not been able to identify a mounting solution that the top elite biathletes find acceptable for high-level competitions. Also, a recent study (Gløersen et al., 2018) where the applied GPS watches were validated indicates sufficient accuracy for our approach. Still, future field-based studies in biathlon should aim to apply higher-accuracy GPS-systems in combination with more advanced sensor technology (e.g., accelerometers and gyroscopes) to gain further knowledge of the competitive demands in biathlon.

### Shooting Performance

The biathletes in this study hit 91 ± 7 and 86 ± 6% of the targets in the prone and standing position, respectively, which is almost equal to the average shooting performance of Top-10 in the World Cup sprint races (Luchsinger et al., 2018). In line with these findings from the biathlon World Cup and Olympic Games, we found a ~5% lower hit rate in the standing compared to the prone position, which was accompanied by a 23% longer penalty time. Thus, most biathletes lose more time due to missed targets in the standing position than in prone in the biathlon sprint competition.

The biathletes' skiing intensities found here are also in line with previous findings of biathlon competitions (Hoffman and Street, 1992). Thus, the biathletes approach the shooting range with similar physiological response as seen 25 years earlier. However, here we found a smaller decrease in %HR_max_ during shooting than reported by Hoffman and Street (1992), which is probably explained by shorter shooting times employed by the biathletes in our study compared to the group of biathletes studied by Hoffmann and Street in 1992. The greater reduction in HR in prone compared to standing position found in this study was also in agreement with Hoffman and Street (1992), which is likely explained by the 13% longer shooting time in prone position in combination with higher HR in upright compared to supine position. The reduction in HR during shooting also puts different demands to the different shots fired in a 5-shot-series and the high HR on the first shot could probably be one reason why biathletes miss the first shot twice as much as the second or third shot during prone shooting in biathlon World Cup sprint races (Luchsinger et al., 2018).

In another study, Hoffman et al. (1992) demonstrated that the exercise intensity negatively influenced parameters related to shooting technique in the standing position but to a lesser extent in prone position. Although no correlation between exercise intensity and shooting performance was seen for any of the shooting positions in our study, this might be explained by the relatively high hit rates and few mistakes to base the statistical tests on. In the future, more detailed analysis of the biathletes' hit points (i.e., measured as distance from center or group diameter) could probably provide additional information on the effect of exercise intensity and the risk of misses at the shooting range. Indeed, Hoffman et al. (1992) showed that hit rate was not affected by exercise intensity, whereas several detailed parameters related to shooting performance such as wobble diameter (movement of the rifle 1 s prior to trigger pull) and the spread of hits (mm) increased with higher exercise intensity, especially in the standing position. Thus, it might be that more detailed analysis of the spread of hits would reveal other relationships to exercise intensity than in our study. Interestingly, it has been suggested that the most important parameters of biathlon shooting technique are movement of the rifle in the vertical direction and cleanness of triggering (i.e., movement of the rifle 0.2–0.0 s before triggering; Ihalainen et al., 2018). This study additionally found that these parameters were negatively affected by high intensity exercise in both junior and elite biathletes, but that elite biathletes scored better than juniors on these technique parameters (Ihalainen et al., 2018). Altogether this indicates that biathletes' shooting technique is altered more by intense exercise than what can be seen on shooting performance measured as number of hits during a competition. This highlights the importance of combining laboratory designed studies with ecologically valid studies measuring actual biathlon race performance. Therefore, further research should examine the relationship between pacing toward shooting and detailed shooting performance (i.e., measure movement of the rifle, spread of hits) through designs that are more experimental in nature.

### Laboratory Determinants Associated With XC Skiing Performance

The predictive values of RPE and %HR_max_ during submaximal roller skiing for XC skiing time and time spent in different terrains were significant for all three sub-techniques. In addition, lower blood lactate concentrations in the G3 and G2 sub-techniques were positively correlated to better XC skiing performance and time spent in downhill terrain. Together, this indicates that the submaximal stages were less demanding for the best performing biathletes in the competition. These findings are in line with a study in cycling, indicating that submaximal measurements based on RPE revealed best relationships both with performance and changes in performance over time (Rodriguez-Marroyo et al., 2017). This highlights the relevance of simply measured variables, such as RPE and %HR_max_, to predict biathlon sprint race performance.

Blood lactate levels on the submaximal stages (performed in uphill terrain) were only correlated to downhill competition performance and not to uphill or varied terrain. This could be explained by the fact that athletes who are faster in the uphill sections are also able to maintain speed better in this terrain and thereby ski faster over hilltops, which subsequently provides higher speeds in the downhill sections. This theory is supported by findings in XC skiing, revealing higher variations in speed at the end of uphill sections and subsequent transition into downhill sections (Andersson et al., 2010), indicating the importance of skiing fast over hilltops in order to create speed in downhills. In addition, a recent study of XC skiers indicate a lag in the physiological response after hilltops and strengthens the importance of being able to create speed at the top of each downhill section (Haugnes et al., 2019). The stronger association between XC skiing performance and RPE or %HR_max_ than the corresponding relationship with blood lactate concentration can likely be explained by the different physiological behavior of these variables; blood lactate concentration might be relatively similar between athletes of different performance levels at low submaximal speed (such as the aerobic steady-state conditions used here), whereas RPE and %HR_max_ would increase more linearly with increased individual load—and thereby be higher in lower level athletes at the same speed.

The submaximal oxygen cost and GE did not correlate to XC skiing performance in this biathlon sprint competition, which is in contrast to studies in the skating technique in XC skiing (Sandbakk et al., 2010, 2011b, 2013). This means that faster biathletes, who are able to ski on a lower %HR_max_ and RPE on the submaximal stages, are not more efficient than lower level biathletes, indicating that additional factors than efficiency would explain the differences in XC skiing performance levels. The most likely explanatory factor is that better XC skiers with similar efficiencies have higher “maximal capacities,” which was also indicated here by the longer TTE of faster skiers. However, VO_2peak_ did not correlate with XC skiing performance in this study, and other factors than measured in our design must have contributed to explain the performance differences in XC skiing both on the field and during the treadmill test. This could be factors such as indices of the anaerobic threshold (which subsequently would allow skiers to compete on a higher fraction of their “maximal capacity”) or anaerobic energy delivery capacity. Therefore, a possible limitation of our approach is the lack of additional physiological measurements (e.g., lactate threshold). The fact that biathletes stop during shooting and have shorter loops of skiing than normally employed in cross-country skiing could imply that biathletes to a greater extent than cross-country skiers must be able to accelerate from more stops during a race and create faster speeds through more turns and smaller but more uphills. However, analyses of the differences between course profiles in XC skiing and biathlon are lacking so the latter assumption remains unknown.

The submaximal stages using different sub-techniques were performed both with and without carrying a rifle, but the order of the stages was not randomized since our purpose was to correlate physiological and perceptual responses with race performance and not to examine the effects of carrying a rifle *per se*. However, the observed correlations did not change when using measurements without carrying a rifle, despite the associated increase in submaximal oxygen cost, HR and blood lactate concentrations compared to roller skiing without the rifle. This indicates relatively robust results in these cases.

Performance in the laboratory, measured as TTE, correlated to overall biathlon sprint race performance, XC skiing time on the last lap and time spent in varied and downhill terrains. When correlated separately with performance on each lap, TTE correlated strongly with performance on the last lap, but not with the first or the second lap. This indicates that better performing athletes in the laboratory adjust their pacing on the two first laps before shooting, which is different from XC skiing, where skiers generally use a more positive pacing than in biathlon. TTE during treadmill roller skiing has also been shown to correlate with on-snow performance in elite XC skiers (Sandbakk et al., 2011a). Hence, this emphasizes the relevance of determining performance by an incremental test to exhaustion in the laboratory when monitoring the development of biathletes' performance level. In addition, it indicates that better performing athletes use different pacing strategies than their lower level peers.

In the current study, no significant associations were identified between VO_2peak_ and XC skiing time, nor time spent in different terrains. These findings are in agreement with Rundell and Bacharach (1995), who showed that TTE during treadmill running and performance during a 1 km double-poling time-trial on snow correlated significantly with performance during a biathlon sprint race among men, whereas VO_2peak_ did not_._ In contrast, previous observations in XC skiing highlight VO_2peak_ as a key determinant of performance (Sandbakk and Holmberg, 2017). The reason for these somewhat conflicting findings are not known but could be explained by differences in the heterogeneity of groups in the different studies or different demands in XC skiing compared to biathlon. However, the average VO_2peak_ among the participants in our study is lower than was previously found in Olympic- or World Championship medalists in biathlon (Tønnessen et al., 2015). In addition, the stages of rifle shooting, leading to periods of ~60–90 s with a reduction in exercise intensity toward shooting in addition to the time spent at the shooting range make biathlon competitions even more interval-based than in XC skiing and pacing strategies may play a more important role.

## Conclusion

The present study showed that XC skiing performance provides greatest impact on overall biathlon sprint performance, with 84% of the variance being explained by this component and most of the variance determined by the time spent in the uphill terrain sections. Overall, this indicates that biathletes should emphasize the development of their XC skiing performance to perform well in biathlon sprint competitions. Although biathletes need to ski fast in all types of terrain, improvements in uphill-specific performance seem to have a particular impact on the overall performance in the biathlon sprint competition. While shooting performance in general is an important component in biathlon, it had clearly lower importance than XC skiing in this study, where penalty time explained 14% of the remaining variance in overall sprint race performance and shooting time and range time together only 2% of the final variance. In addition, race tactics and pacing are important aspects in biathlon competitions and our data indicate a further potential to enhance performance by optimizing pacing strategies, especially among the slower skiers who lose most time on the last lap to the faster skiers.

Based on treadmill roller ski tests in the laboratory, lower ratings of RPE and %HR_max_ during submaximal roller skiing in the three main sub-techniques of skating, as well as longer TTE during an incremental test to exhaustion, were strongly correlated to overall biathlon sprint performance and XC skiing time. Such laboratory-derived measures may therefore be validly used to distinguish biathletes of different performance levels and to track progress of their XC skiing capacity. In contrast, the non-significant relationships to peak oxygen uptake and gross efficiency indicate that other variables than those measured here (such as anaerobic capacity) also contributed to TTE and biathlon performance in these athletes.

## Data Availability Statement

The datasets generated for this study are available on request to the corresponding author.

## Ethics Statement

Ethical review and approval was not required for the study on human participants in accordance with the local legislation and institutional requirements. The patients/participants provided their written informed consent to participate in this study.

## Author Contributions

All authors contributed in the design of the study. HL and RT collected the data. JK performed the data handling of GPS and HR-signals. RT performed the statistical analyses. All authors contributed to the writing of the manuscript and to the design of figures and tables.

### Conflict of Interest

The authors declare that the research was conducted in the absence of any commercial or financial relationships that could be construed as a potential conflict of interest.
